# ^68^Ga-PSMA-11 PET/CT for prostate cancer staging and risk stratification in Chinese patients

**DOI:** 10.18632/oncotarget.14691

**Published:** 2017-01-17

**Authors:** Shiming Zang, Guoqiang Shao, Can Cui, Tian-Nv Li, Yue Huang, Xiaochen Yao, Qiu Fan, Zejun Chen, Jin Du, Ruipeng Jia, Hongbin Sun, Zichun Hua, Jun Tang, Feng Wang

**Affiliations:** ^1^ Department of Nuclear Medicine, Nanjing First Hospital, Nanjing Medical University, Nanjing 210006, China; ^2^ Department of Nuclear Medicine, PET Centre, No. 1 Hospital Affiliated to Nanjing Medical University, Nanjing 210029, China; ^3^ Department of Pathology, Nanjing First Hospital, Nanjing Medical University, Nanjing 210006, China; ^4^ Department of Nuclear Medicine, The Affiliated Jiangyin Hospital of Southeast University Medical College, Jiangyin 214400, China; ^5^ Department of Technology Development, China Isotope Radiation Corporation, No. 1 Nansixiang, Sanlihe, West District, Beijing 100045, China; ^6^ Department of Urology, Nanjing Medical University, Nanjing 210006, China; ^7^ The State Key Laboratory of Pharmaceutical Biotechnology, Department of Biochemistry, College of Life Sciences, Nanjing University, Nanjing 210006, China; ^8^ Department of Nuclear Medicine, The Second Affiliated Hospital of Soochow University, Suzhou 215004, China

**Keywords:** treatment-naïve prostate cancer, prostate-specific membrane antigen, metastatic castrate-resistant prostate cancer, staging, risk stratification

## Abstract

We evaluated the clinical utility of ^68^Ga-PSMA-11 PET/CT for staging and risk stratification of treatment-naïve prostate cancer (PCa) and metastatic castrate-resistant prostate cancer (mCRPC). Twenty-two consecutive patients with treatment-naïve PCa and 18 with mCRPC were enrolled. ^68^Ga-PSMA-11 PET/CT and magnetic resonance imaging (MRI) were performed for the evaluation of primary prostatic lesions, and bone scans were used for evaluation bone metastasis. Among the 40 patients, 37 (92.5% [22 treatment-naïve PCa, 15 mCRPC]) showed PSMA-avid lesions on ^68^Ga-PSMA-11 images. Only 3 patients with stable mCRPC after chemotherapy were negative for PSMA. The sensitivity, specificity and accuracy of ^68^Ga-PSMA-11 imaging were 97.3%, 100.0% and 97.5%, respectively. The maximum standardized uptake (SUV_max_) of prostatic lesions was 17.09 ± 11.08 and 13.33 ± 12.31 in treatment-naïve PCa and mCRPC, respectively. ^68^Ga-PSMA-11 revealed 105 metastatic lymph nodes in 15 patients; the SUV_max_ was 16.85 ± 9.70 and 7.54 ± 5.20 in treatment-naïve PCa and mCRPC, respectively. ^68^Ga-PSMA-11 PET/CT also newly detected visceral metastasis in 9 patients (22.5%) and bone metastasis in 29 patients (72.5%). ^68^Ga-PSMA-11 PET/CT exhibits potential for staging and risk stratification in naïve PCa, as well as improved sensitivity for detection of lymph node and remote metastasis.

## INTRODUCTION

Prostate cancer (PCa) is the most common solid neoplasm in men and the second leading cause of cancer-related death in men in Europe and the United States [1, 2]. Early detection of localized disease results in a five-year survival rate of nearly 100%. On the other hand, tumor metastasis leads to dramatically reduced survival rates. Prostate-specific antigen (PSA) screening and active surveillance are helpful, but may lead to over-diagnosis and overtreatment, as the majority of PCa are indolent and progress very slowly [3]. Consequently, precise characterization is critical for appropriate clinical management of PCa.

In China, the incidence of PCa is increasing, in part due to lifestyle changes during the last decade. Up to now, however, the epidemiology of PCa in China has been unclear, as have the phenotypes and heterogeneity among Chinese patients [4–6]. PSA screening system has not been established, and a majority of patients are already at an advanced stage at their initial diagnosis. Radical prostatectomy is not an option, leaving androgen-deprivation therapy as the main strategy in the management of PCa. Although a majority of PCa patients respond to androgen suppression, initially, they eventually progress to castration-resistant prostate cancer (CRPC) [7, 8].

Recent advances in magnetic resonance imaging (MRI) of the prostate gland have improved our ability to detect and stage prostate adenocarcinoma, and multiparametric MRI further facilitated clinical risk assessment and predicting outcomes in prostate cancer [9]. Moreover, MRI/TRUS biopsy is now recommended by the American Urological Association for patients undergoing repeated prostate biopsies [10]. However, MRI may miss up to 20% of clinically significant cancers; it has low sensitivity for the detection of lymphadenopathy, and specificity is also limited [11–14]. Prostate adenocarcinoma in the peripheral zone shows low signal intensity that is easily distinguished from the normal high-signal peripheral zone, but the low signal intensity is nonspecific and may also be seen in benign conditions, such as prostatitis and post-radiation fibrosis.

Molecular imaging in PCa is continuously evolving in parallel with greater understanding of the underlying biological heterogeneity, which is characteristic of the disease and is critical for risk stratification and selection of the treatment strategy. ^11^C-choline, 1-amino-3-^18^F-fluorocyclobutyl-1-carboxylic acid [^18^F-FACBC]) and ^223^Ra-dichloride radionuclide therapies have been approved by the U.S. Food and Drug Administration for clinical use in PCa [15–17]. In addition, PET/CT with sodium fluoride is now being utilized clinically and shows greater sensitivity for detection of bone metastasis [18]. However, ^18^F- and ^11^C-labeled choline have low sensitivity and specificity, especially in patients with low PSA levels [19–22].

Prostate-specific membrane antigen (PSMA), also known as folate hydrolase I or glutamate carboxypeptidase II, is an outstanding target for imaging and treatment [23]. PSMA is overexpressed on prostatic cancer cell surfaces, including advanced-stage prostate carcinoma cells, and in the tumor neovasculature, but is weakly expressed in normal prostate tissues [24–26]. PSMA expression is further increased in patients with metastatic disease and/or those who have progressed to a hormone-refractory state [27,28]. PSMA levels increase with upstaging and increased tumor grade [29–32]. Moreover, long-term androgen-depriving therapy may increase tumoral PSMA expression, thereby facilitating metastasis. In the last two years, increases in the sensitivity and specificity of ^68^Ga- and ^18^F-labeled small-molecule PSMA inhibitors has enabled improved staging and detection of recurrence and metastasis in large numbers of patients. Furthermore, the ability of PSMA PET to localize recurrence in patients with rising PSA levels appears to be markedly superior to other clinically available imaging [33–35]. In this prospective study, the clinical utility of [^68^Ga]Glu-urea-Lys(Ahx)-HBED-CC (^68^Ga-PSMA-11) PET/CT for staging and risk stratification of treatment-naïve PCa was validated. The diagnostic value for patients with metastatic castrate-resistant prostate cancer (mCRPC) was also addressed. To our knowledge, this is the first report on the use of ^68^Ga-PSMA-11 for the diagnosis of PCa in a Chinese population.

## RESULTS

### Patient characteristics

Among the 40 consecutive patients were included in this study (Table 1), the median patient age was 74 years (range, 53-91 years). Median serum PSA level at the time of imaging was 117.05 ng/ml (range 0.01-12,356.00 ng/ml). Among the 18 patients with mCRPC, PSA levels ranged from 4.05 to 12,356 ng/ml (2384.95 ± 4135.96 ng/ml), whereas PSA levels ranged from 0.04 to 7,890 ng/ml (1247.57 ± 2150.77 ng/ml) among treatment-naïve PCa patients. The median Gleason score was 8 (range, 6-9).

**Table 1:** Patient characteristics

Characteristics	
**Age, years**	74 (53-91)
**Gleason score**	8 (6-9)
**Initial PSA level, ng/ml**	117.05 (0.01-12356.0)
**Time since diagnosis**	1 week to 9 years
**Metastatic lesions**	
**Bone metastasis**	109 lesions in 29 patients
**Lymph node metastasis**	105 lesions in 15 patients
**Liver metastasis**	9 lesions in 4 patients
**Lung metastasis**	6 lesions in 5 patients

Data are expressed as the number or the median (range).

Pathology and clinical follow-up confirmed there were 29 patients with bone metastasis, 15 with lymph node metastasis, 4 with liver metastasis, and 5 with lung metastasis (for the SUV_max_ of the metastases, see Table 2). Radical prostatectomy was performed on four patients with localized PCa, who exhibited no abnormal ^68^Ga-PSMA-11 uptake, except in the primary prostatic lesion. In 17 patients with treatment-naïve PCa, more lesions were found using ^68^Ga-PSMA-11 uptake than CT or MRI. Those patients were upstaged and avoided radical prostatectomy.

**Table 2:** SUV_max_ of primary prostatic lesion and metastasis on ^68^Ga-PSMA-11 images

Lesions	SUV_max_
**Primary prostatic lesion (whole)**	15.87 ± 11.43
**Diagnosed treatment-naïve PCa**	17.09 ± 11.08
**mCRPC**	13.44 ± 12.31
**Lymph node metastasis**	14.54 ± 9.66
**Bone metastasis**	27.57 ± 19.71
**Lung metastasis**	5.07 ±1.83
**Liver metastasis**	20.63 ± 10.92

Data are expressed as the mean ± SD.

PCa: prostate cancer, mCRPC: metastatic castration-resistant prostate cancer.

### ^68^Ga-PSMA-11 image interpretation

No adverse events were observed in this study. The biodistribution of ^68^Ga-PSMA-11 was favorable, with only mild or moderate uptake into the salivary glands and small intestine (Supplementary Table 1). The SUV_max_ in the salivary glands, small intestine, liver, kidneys, and lungs was 14.42 ± 5.91, 9.47 ± 4.99, 3.99 ± 2.11, 36.06 ± 11.7, and 11.32 ± 0.53, respectively. High-contrast images were obtained, even with a low dose of ^68^Ga-PSMA-11 (135.67 MBq). Prostatic primary lesions and metastatic sites showed significantly greater uptake, and T/NT ratios ranged from 1.54 to 207.85; the highest T/NT ratios were at sites of bone metastasis (45.95 ± 45.78) and lymphadenopathy (13.85 ± 9.20) (Supplementary Figure 1). All 22 treatment-naïve PCa patients had PSMA-avid lesions. Of the 18 patients with mCRPC, 15 had PSMA-avid lesions.

### Staging and clinical management

### Primary prostatic lesion

**Figure 1:**
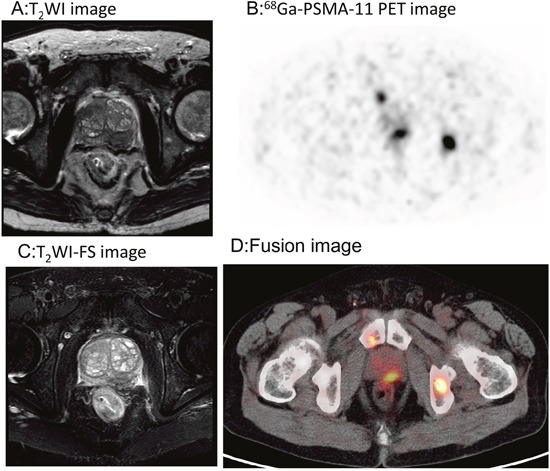
68Ga-PSMA-11PET/CT versus MRI for the evaluation of the primary prostatic lesions Focal uptake was found in the left peripheral zone of the prostate gland, which matched the hypointensity signal on MRI. **A**. T_2_WI image. **B**. ^68^Ga-PSMA-11 PET transverse image. **C**. T_2_WI-FS image. **D**. Fusion image.

In treatment-naïve PCa patients (Gleason score 6-9), all prostatic primary lesions were PSMA-avid on ^68^Ga-PSMA-11 images (SUV_max_ 17.09 ± 11.08). In 4 patients, no extraprostatic lesions were found. One patient who was scheduled with local radical prostatectomy had 3 pelvic lymph nodule metastases in addition to his prostatic lesion. Ten patients with mCRPC were administered androgen depriving and chemotherapy. These patients were not treated with radical prostatectomy due to their advanced stage, with multiple lymph node and bone metastases. The prostatic primary lesions were visualized using high focal uptake (SUV_max_ 13.33±12.31, n=10). Pelvic MRIs showed a hypo-intensity T2WI signal in the peripheral zone of the cancerous prostate, and some patients exhibited invasion of peripheral organs, including the seminal vesicles.^68^Ga-PSMA-11 PET/CT enabled precise definition of the prostatic primary lesion and was consistent with the MRI findings. A representative case is shown in Figure 1.

### Lymphadenopathy and remote metastasis

**Figure 2:**
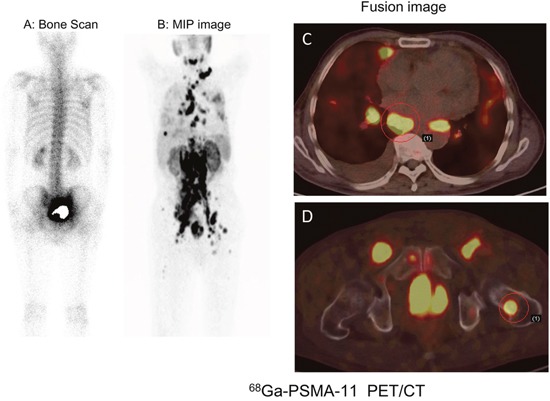
68Ga-PSMA-11 PET/CT versus bone scan for the evaluation of multiple metastases in a 73-year-old patient with right leg edema Besides the primary prostatic lesion, multiple PSMA-avid lesions in lymph node and bone metastases were detected on ^68^Ga-PSMA-11 images. **A**. Bone scan. **B, C, D**. ^68^Ga-PSMA-11 images: B, MIP image; C, thoracic fusion image; D, Pelvic fusion image.

In the 15 patients with lymph node metastasis, ^68^Ga-PSMA-11 PET/CT revealed a total of 105 metastatic lymph nodes, with SUV_max_ values of 14.54 ± 9.66 and sizes ranging from 3.0 to 33.5 mm (9.96 ± 6.21 mm). The SUV_max_ of the metastatic nodes was 16.85 ± 9.70 and 7.54 ± 5.20 in treatment-naïve PCa and mCRPC, respectively. Eight patients with treatment-naïve PCa showed metastasis to multiple lymph nodes, including the mediastinal, supraclavicular, subclavicular, and hilar nodes. A representative case is shown in Figure 2. It is noteworthy that 2 patients, multiple lymph node metastases and showing iliac lymph node involvement exhibited limb edema. In addition, lymph nodule enlargement was associated with compression of ureter in 1 patient, which led to obstruction and hydronephrosis.

**Figure 3:**
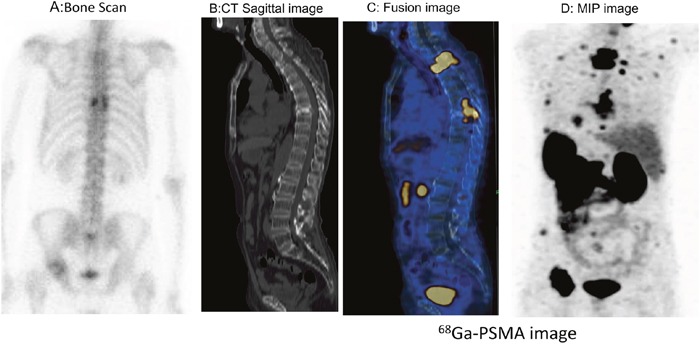
Thoracic spinal cord invasion was detected on 68Ga-PSMA-11 PET/CT images in a patient with mCRPC Multiple PSMA-avid lesions in the thoracic spine, ribs and pelvis were also detected. The spinal cord was also involved (SUV_max_ = 110). **A**. Bone scan. **B**. CT image. **C, D**. ^68^Ga-PSMA-11 PET /CT images: C, fusion image; D, MIP projection.

**Figure 4:**
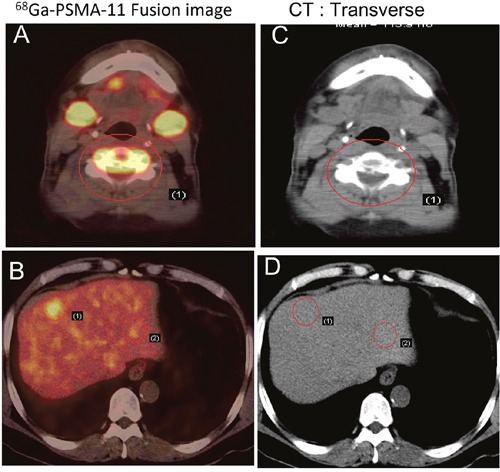
Early detection of PCa metastasis on 68Ga-PSMA-11 PET/CT images Two years after androgen-deprivation therapy, a patient with mCRPC showed elevated PSA (5.2 ng/ml) and moderate uptake in the fifth cervical vertebra on a bone scan. PSMA avid lesions were at C5 and in the liver. **A, B**. 68Ga-PSMA-11 PET/CT fusion image. **C, D**. CT image.

**Figure 5:**
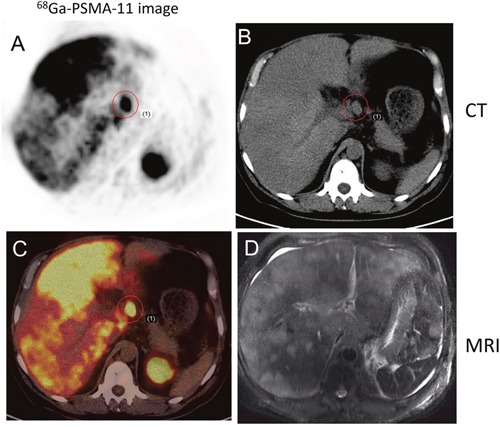
68Ga-PSMA-11 PET/CT versus T2WI MRI in a 52-year-old patient with progressive disease PCa was confirmed by lymph node aspiration; the serum PSA level was >10,000 ng/ml at the time of imaging. ^68^Ga-PSMA-11 PET/CT detected multiple lymph node and liver metastases. **A**. Transverse image. **B**. CT. **C**. Multiple sites of focal uptake in the liver. **D**. MRI.

In 29 patients with bone metastasis, SUV_max_ ranged from 3.00 to 124.71 (27.57 ± 27.46). In 3 patients with stable disease, the PSA values ranged from 0.01 to 0.07 ng/ml, and no ^68^Ga-PSMA-11 uptake was observed, including into osteoblastic bone lesions. Of the 22 patients with treatment-naïve PCa, 17 had multiple lymph node and bone metastases. Based on these findings, these patients initially received hormonal treatment and radiotherapy or chemotherapy. For those with only pelvic lymph node positivity, radiotherapy was considered depending on the magnitude of PSA decline after hormone deprivation. In one patient with spinal cord involvement (SUV_max_ = 124.71), multiple lymph node and bone metastases were found in ^68^Ga-PSMA-11 images (Figure 3). In addition, ^68^Ga-PSMA-11 PET/CT newly detected lung metastasis in 5 patients and liver metastasis in 4 patients (Figures 4 and 5). In total, 17 patients with treatment-naïve PCa were upstaged after ^68^Ga-PSMA-11 PET/CT.

### PSMA expression

**Figure 6:**
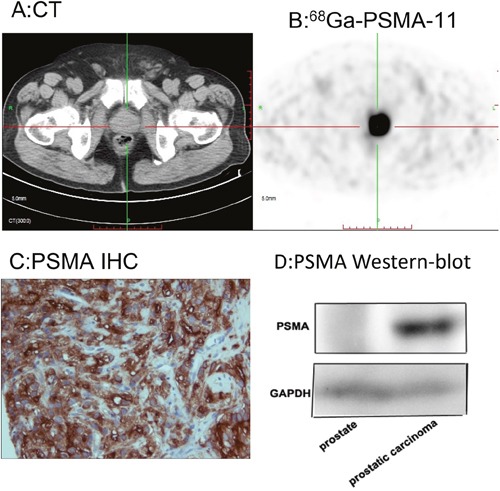
68Ga-PSMA-11 PET/CT enables quantitation of PSMA expression in the primary lesion, which was consistent with histopathological analysis ^68^Ga-PSMA-11 PET/CT showed high focal uptake in the primary prostatic lesion. **A**. CT image. **B**. Fusion image. **C**. PSMA immunostaining. **D**. Western blot.

Immunohistochemistry confirmed that PSMA was significantly overexpressed in the primary prostatic lesions, which was consistent with the PSMA-avid lesions in ^68^Ga-PSMA-11 images. This finding was further confirmed in western blot and immunofluorescence analyses (Figure 6 and Supplementary Figure 2).

## DISCUSSION

^68^Ga- and ^18^F-labeled small-molecule PSMA inhibitors previously showed potential utility for detection of metastatic spread of recurrent PCa [36–37]. Further large-scale clinical studies validated the exceptionally high accuracy of this approach for detection of recurrent prostate cancer [38–39]. In the present prospective study, ^68^Ga-PSMA-11 PET/CT accurately localized the primary prostatic lesion and metastasis in treatment-naïve prostate cancer patients, and significantly improved detection rate of the malignant lesions in patients with mCRPC, even when PSA levels were low.

^68^Ga-PSMA-11 showed high sensitivity for the detection of primary prostatic lesions. All primary lesions in treatment-naïve prostate cancer patients showed high focal uptake with 100% sensitivity. In 10 patients with mCRPC, who did not receive radical prostatectomy, ^68^Ga-PSMA-11 PET/CT also revealed a PSMA-avid lesion in the prostate gland. With the use of endorectal coils, MRI has become an essential tool for localization of the primary lesion and accurate staging of PCa, especially T staging [40]. However, the low specificity of MRI limits its clinical value [41]. By contrast, ^68^Ga-PSMA-11 PET/CT combined with MRI could be used for T staging, enabling accurate evaluation of T staging of prostate cancer, which would have great impact on clinical management.

^68^Ga-PSMA-11 PET/CT also showed high sensitivity for detection of metastatic lymph nodes. A total of 105 lymph node metastases, ranging in size from 3 to 35.5 mm, were detected using ^68^Ga-PSMA-11. By contrast, CT and MRI have low sensitivity for lymph node metastasis, with detection only of lesions >10 mm in diameter. Consequently, CT and MRI may fail to detect microscopic invasion of the lymph nodes [42,43]. In the present study, multiple lymph node involvements affecting the mediastinum, retroperitoneum and pelvis were detected in 8 patients using ^68^Ga-PSMA-11 PET/CT, but were negative on CT and/or MRI. Thus, ^68^Ga-PSMA-11 PET/CT has some merit for N staging in prostate cancer. It is noteworthy that iliac lymph node involvement may lead to limb edema and ureter obstruction and hydronephrosis. Thus if a male patient presents with limb edema or hydronephrosis, prostate cancer with iliac lymph node metastasis should be suspected.

In this study, ^68^Ga-PSMA-11 PET/CT revealed visceral metastasis in 9 patients (22.5%), lung metastasis in 5 patients, and liver metastasis in 4 patients. This incidence of visceral metastasis is significantly higher than previously reported [44], in large part because liver and lung metastases have traditionally been relatively rare events even in mCRPC. The high incidence of visceral metastasis in the present study may be related to the unique behavior of PCa in Chinese ethnics. The distribution of metastases in advanced prostate cancer is prognostic. Visceral involvement, especially liver and lung metastasis, associates with increased disease lethality, and patients with visceral metastasis have poorer survival than those with bone metastasis and nonvisceral involvement [44]. ^68^Ga-PSMA-11 PET/CT was valuable for localization of less common metastatic lesions that were not visible on conventional images.

**Figure 7:**
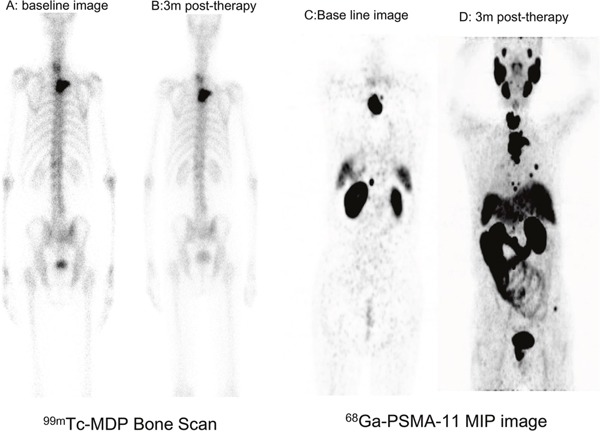
Serial 68Ga-PSMA-11 images in a case with progressive mCRPC **A, B**. Bone scan showing no changes in a patient with mCRPC after ADT therapy. **C, D**. ^68^Ga-PSMA-11 PET/CT revealed multiple metastases and progression compared to a baseline image.

Bone scans have little diagnostic value for cervical vertebral lesions. ^68^Ga-PSMA-11 PET/CT improved the sensitivity for detection of affected cervical vertebrae and rare metastases, such as those of the head and distal extremities. Bone metastasis showed the highest uptake in ^68^Ga-PSMA-11 images, the ratio of metastatic bone lesion to normal bone tissue was high (Figure 7). Hematogenous metastasis via the vertebral venous plexus is the primary metastatic pathway of PCa, and ^68^Ga-PSMA-11 PET/CT also exhibited high sensitivity for the detection of spinal cord invasion. PSMA-based therapy provides an alternative strategy to treatment of bone metastasis, and it provides guidance for bone-seeking radiotracer therapy with ^89^Sr and ^223^Ra. If, however, spinal cord invasion is confirmed, radionuclide therapy is contraindicated. Thus, ^68^Ga-PSMA-11 PET/CT has utility for M staging, and can be used for risk stratification and selection of treatment strategy.

PSMA was significantly overexpressed in primary prostatic lesions. Use of ^68^Ga-PSMA-11 could enable quantification of tumoral PSMA expression, which would be useful for selection of biopsy sites in patients with unknown primary lesions, and might even be critical for PSMA-based theranostics.

This small cohort study demonstrated that ^68^Ga-PSMA-11 PET/CT has high sensitivity and specificity for localization of primary prostatic lesions and metastatic sites, and enabled precise staging and risk stratification in patients with treatment-naïve PCa. ^68^Ga-PSMA-11 PET/CT also showed high sensitivity for detection of metastasis and recurrence in patients with mCRPC, even when PSA levels were low. The sensitivity was much higher than in other studies, likely due to the presence of more extensive metastatic disease and higher Gleason scores in our patient cohort, or perhaps to biological factors unique to Chinese ethnics. ^68^Ga-PSMA-11 PET/CT played an important role in staging and detection of remote and uncommon metastatic sites. This imaging modality might therefore be effectively utilized for evaluation of biological behavior and risk stratification in PCa. Our understanding of what drives the development of different metastatic patterns in PCa is limited. ^68^Ga-PSMA-11 PET/CT has the potential to shed new light on the mechanisms underlying PCa metastasis and to serve as the basis for development of novel treatment approaches for Chinese men with prostate carcinoma.

## MATERIALS AND METHODS

A total of 40 patients with PCa (22 patients with biopsy-proven, naïvely diagnosed PCa and 18 patients with mCRPC) were prospectively enrolled in this study conducted at Nanjing First Hospital from January to December 2015. All patients gave written informed consent. All reported investigations were conducted in accordance with the Declaration of Helsinki and with our national regulations. All patients underwent PET/CT 1 h after injection of ^68^Ga-PSMA-11. MRI was performed to evaluate the primary prostatic lesion and peripheral invasion, and a ^99m^Tc-MDP bone scan was performed to evaluate bone metastasis. Gleason scores were calculated based on aspiration biopsies or surgery samples.

### Preparation of ^68^Ga-PSMA-11 and quality control

^68^Ga-PSMA-11 was prepared with an ITG semi-automated module (Germany, Munich). Radiochemical purity and stability were determined using analytical reverse phase high-performance liquid chromatography. ^68^Ga-PSMA-11 was stable *in vitro*, its radiochemical purity was >99% after 2 h of radiolabeling. All products were prepared using good manufacturing practices and were nonpyrogenic and sterile.

### Imaging protocol

All patients underwent PET/CT in a Biograph HR scanner (Siemens, Erlangen, Germany) 60 min after intravenous injection of ^68^Ga-PSMA-11 (median, 131.72 MBq, range 130.6-177.6 MBq). First, a CT scan (130 keV, 80 mAs) was obtained without using contrast medium. Static emission scans, corrected for dead time, scatter and decay, were acquired from the vertex to the proximal legs. This required the patient assume 8 bed positions with 3 min per bed position. The images were iteratively reconstructed and included CT-based attenuation correction with the OSEM algorithm using 4 iterations with 8 subsets and Gaussian filtering to an in-plane spatial resolution of 5 mm at full-width at half-maximum. For calculation of the standardized uptake value (SUV), circular regions of interest were drawn around the area with focally increased uptake in transaxial slices and automatically adapted to a three-dimensional volume of interest using e.soft software (Siemens) at 50% isocontour. If a patient was suspected of having metastasis to the head or extremities, a head-to-toe protocol was used, with identical parameters for all patients. Datasets were fully corrected for random coincidences, scatter radiation, and attenuation. Corrected images were assessed clinically by certified nuclear medicine physicians.

MRI was performed using an eight-channel torso phase-array coil at 3 Tesla (Phillips, Gyroscan ACS-NT). In brief, T1-and T2-weighted sequences and diffusion-weighted images (DWI) were acquired. Pelvic assessment and lymph node staging was accomplished using 5-mm T2W turbo spin echo (TSE) transverse and coronal short-tau inversion recovery (STIR) sequences. For the prostate gland, 3-mm endorectal T2W spin echo (SE) sagittal, transverse, and coronal sequences were acquired. A reference slice was defined in the central part of the organ, measured from the prostate base. All patients had T1WI, T2WI, and T2WI-FS scans in three planes (transverse, coronal, and sagittal).

### PET/CT image interpretation

The physiologic activities of ^68^Ga-PSMA-11 in the parotid and submandibular glands as well as organs such as lung, liver, spleen, bowel, and kidney were determined and expressed as the maximum standardized uptake value (SUV_max_) on images obtained 1 h post-injection. Images were interpreted by three experienced nuclear medicine physicians based on visual assessment. Final decisions were reached by consensus. A positive scan was defined as one showing abnormal focal increases in tracer activity within a lesion with an intensity level higher against a surrounding background considered to be malignant. Any hilar or mediastinal lymph node activity greater than the mediastinal activity was regarded as an abnormal. Any distant focally increased tracer activity that did not correspond to a normal physiological structure was considered to be a metastatic lesion. The malignant lesion to normal tissue (T/NT) ratio was calculated based on ratio of the SUV_max_ of the lesions to the contralateral normal tissue.

### Anatomical image interpretation

The initial staging of PCa was based on findings from conventional imaging techniques, including ultrasonography, enhanced CT and MRI, bone scans, and fine needle aspiration biopsies when required/needed. Any sign of enlargement of the prostate gland, heterogeneous density, small patchy calcifications, and/or invasion of peripheral organs on CT, was regarded as abnormal. Any sign of heterogeneous signal intensities, signal hypointensity in the peripheral zone, invasion of the prostate capsule or seminal vesicle, or enlarged pelvis lymph nodes on MRI was regarded as a cancerous lesion. Considering lymphadenopathy, a lymph node were accepted as pathologic if the short axis diameter was >1.0 cm on transaxial CT or MRI images.

### PSMA expression in the primary prostatic lesion: immunohistochemistry and immunofluorescent staining

Tissue samples were fixed with 5% formaldehyde, embedded in paraffin, and cut into consecutive 5 μm sections using standard procedures. The sections were incubated first with primary anti-PSMA monoclonal antibody overnight at 4°C. Then after three washes with PBS, sections were incubated with horseradish peroxidase (HRP)-labeled secondary antibody for 1 h at room temperature. The bound antibody complexes were detected using a diaminobenzidine detection system, after which the slides were counterstained with hematoxylin. Alternatively, for immunofluorescent staining, sections were incubated with a fluorescently labeled secondary antibody for 1h at room temperature in the dark, after which nuclei were counterstained using 4, 6-diamidino-2-phenylindole. Immunostained images were observed with microscope (Leica DM IL).

### Statistical analysis

The sensitivity, specificity, and accuracy of each imaging modality were calculated. Descriptive data are presented as the mean and standard deviation (SD) for normally distributed variables. Paired Student's *t*-test was used to evaluate differences among normally and non-normally distributed variables. Values of p < 0.05 were considered significant. SPSS 21.0 software was used for analysis.

## SUPPLEMENTARY MATERIALS FIGURES AND TABLES


