# Supplementary material for: 68Ga-PSMA-11 PET/CT for prostate cancer staging and risk stratification in Chinese patients
Source: Oncotarget. 2017 Jan 17;8(7):12247–58. doi: 10.18632/oncotarget.14691 (PMC5355341; doi:10.18632/oncotarget.14691)
Supplement: Supplementary file 1 [file oncotarget-08-12247-s001.pdf]

^{68}Ga-PSMA-11 PET/CT for prostate cancer staging and risk stratification in Chinese patients

SUPPLEMENTARY FIGURES AND TABLE

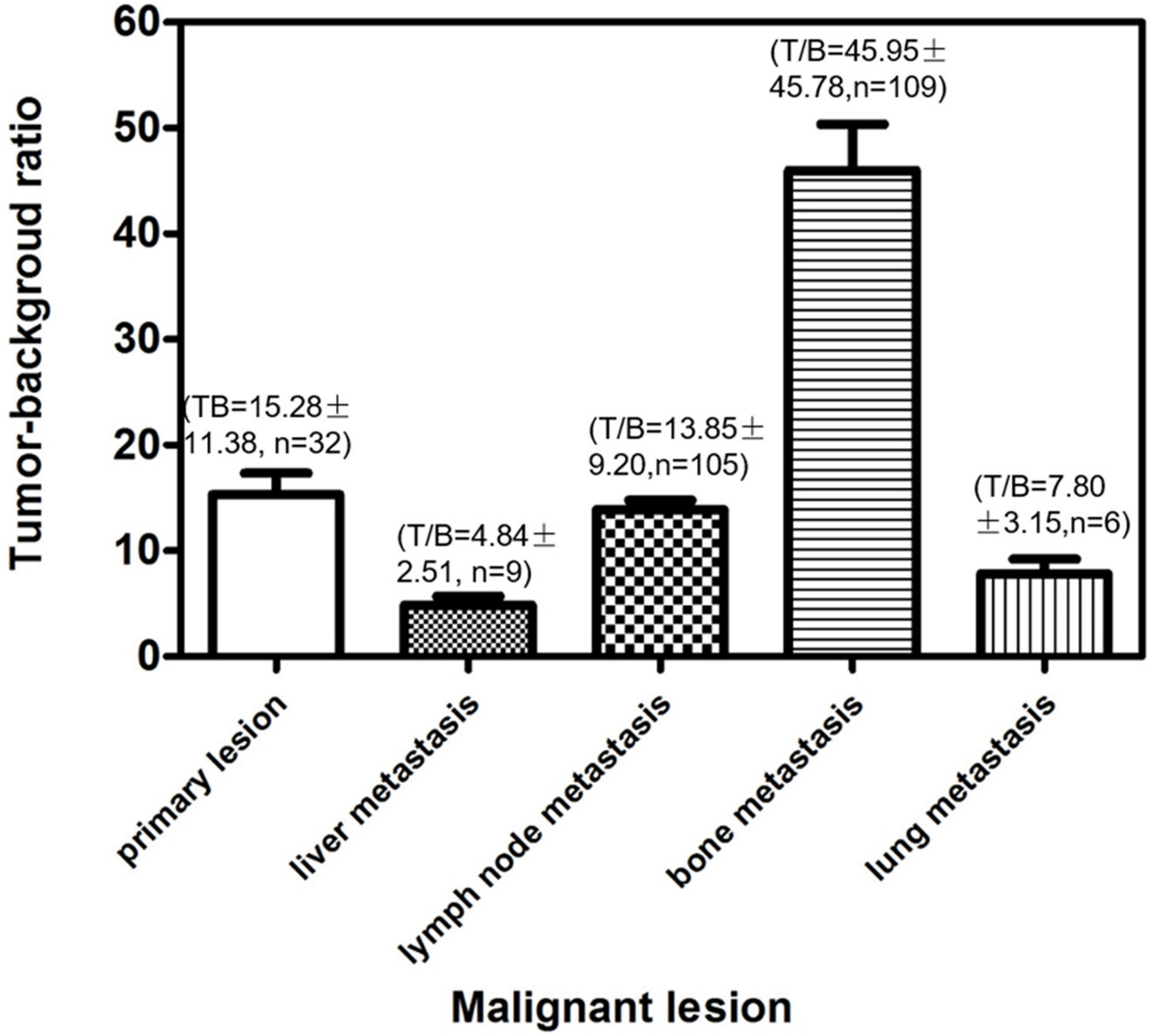

Supplementary Figure 1: Tumor to normal tissue ratio in a primary prostatic lesion and remote metastasis. ^{68}Ga-PSMA-11 PET/CT showed higher tumor to normal tissue ratio not only in the primary prostatic lesion but also in metastatic sites. Tumor to normal tissue ratio was highest in bone metastasis, followed by lymph node metastasis.

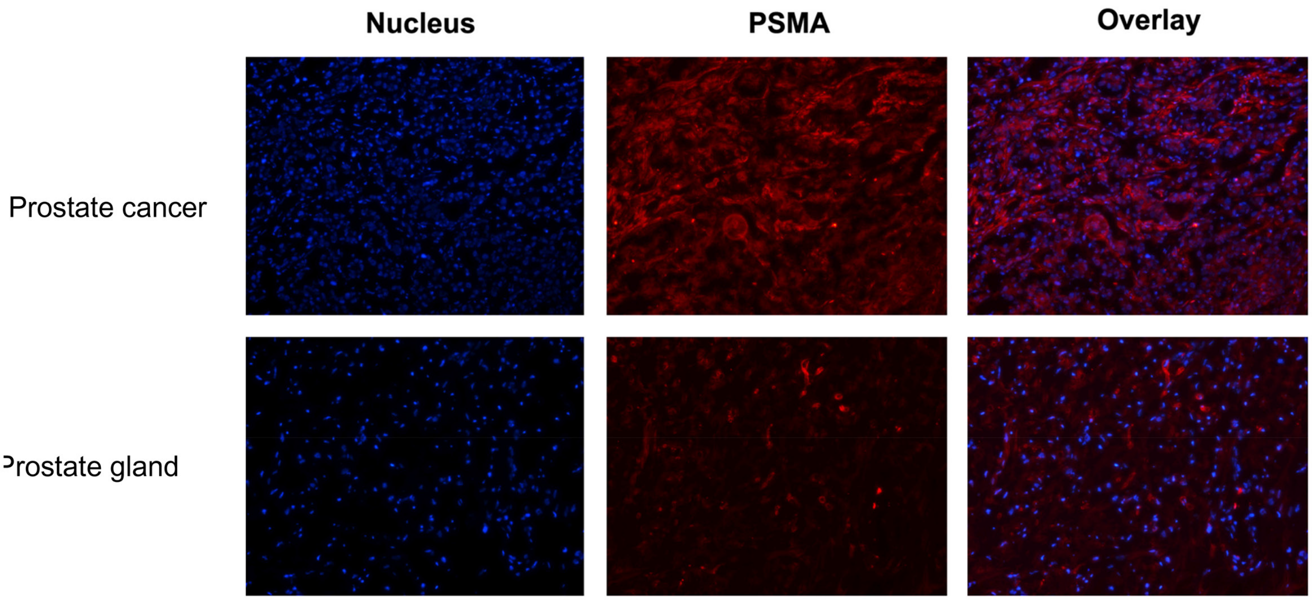

Supplementary Figure 2: Significant PSMA expression was confirmed by immunofluorescence imaging in a patient with diagnosed treatment-naïve PCa.

Supplementary Table 1: ^{68}Ga-PSMA-11 biodistribution in visceral organs

Organs	SUV_{max}
**Salivary gland**	14.42 ± 5.91
**Small intestinal**	9.47 ± 4.99
**Liver**	3.99 ± 2.11
**Kidney**	36.06 ± 11.71
**Lung**	1.32 ± 0.53

SUV_{max}: maximum standardized uptake value.
